# Pathological complete response, histologic grade, and level of stromal tumor-infiltrating lymphocytes in ER + HER2- breast cancer

**DOI:** 10.1186/s13058-025-01999-7

**Published:** 2025-03-20

**Authors:** Seung Ho Baek, Min Ji Lee, Yoonwon Kook, Soong June Bae, Joon Jeong, Yoon Jin Cha, Sung Gwe Ahn

**Affiliations:** 1 Department of Surgery, Yongin Severance Hospital, Yonsei University College of Medicine, Yongin, Republic of Korea; 2 Department of Surgery, Gangnam Severance Hospital, Yonsei University College of Medicine, Seoul, Republic of Korea; 3 Department of Pathology, Gangnam Severance Hospital, Yonsei University College of Medicine, Seoul, Republic of Korea; 4 Institute for Breast Cancer Precision Medicine, Yonsei University College of Medicine, Seoul, Republic of Korea

**Keywords:** Immune checkpoint inhibitors, Neoadjuvant chemotherapy, Estrogen receptor-positive, Human epidermal growth factor receptor 2-negative, Pathological complete response, Stromal tumor-infiltrating lymphocytes, Histologic grade

## Abstract

**Background:**

Recent trials have integrated immune checkpoint inhibitors (ICIs) into neoadjuvant chemotherapy (NAC) in patients with estrogen receptor (ER)-positive, human epidermal growth factor receptor 2 (HER2)-negative breast cancer of histologic grade (HG) III. We assessed the pathological complete response (pCR) rate according to the level of stromal tumor-infiltrating lymphocytes (sTIL) and HG in patients with ER + HER2- breast cancer undergoing NAC.

**Methods:**

Between January 2016 and December 2023, we retrospectively identified 376 patients with ER + HER2- breast cancer who underwent NAC followed by surgery. HG and sTIL levels were examined in the biopsied samples before NAC. Multiple sTIL cutoff values as 10%, 20%, and 30% were applied.

**Results:**

Twenty-seven patients (7.2%) had HG III tumors. The pCR rate in the HG III group was 22.2%, which was significantly higher than that in the HG I/II group (4.0%) (*p* < 0.001). The HG III group had a higher mean sTIL level than HG I/II group (38.7% vs. 12.9%; *p* < 0.001). According to the sTIL levels, the pCR rate in the high sTIL group was significantly higher than that in the low sTIL group: i) cutoff of 10%, 2.4% vs. 9.5%; cutoff of 20%, 2.8% vs. 13.7%; and cutoff of 30%, 3.2% vs. 18.3%. In the high sTIL (≥ 30%) group, the pCR rate for HG III was 33.3%, whereas that for HG I/II was 13.3%.

**Conclusions:**

High tumor grade and sTIL levels were associated with higher rates of pCR in ER + HER2- breast cancer. Our findings support that the addition to ICIs to NAC increased pCR in high-risk, HG III, ER + HER2- breast cancer and suggest that sTIL levels could be utilized to identify patients with ER + HER2- breast cancer eligible for chemoimmunotherapy.

**Supplementary Information:**

The online version contains supplementary material available at 10.1186/s13058-025-01999-7.

## Introduction

The use of neoadjuvant chemotherapy (NAC), which involves the administration of systemic chemotherapy before surgery, is increasing in the treatment of breast cancer. NAC provides the advantage of reducing tumor size and axillary nodal burden, thereby minimizing the extent of surgery [1–4]. Additionally, several studies have shown that patients who achieve pathological compete response (pCR) after NAC have a better prognosis than those with residual disease in human epidermal growth factor receptor 2 (HER2)-positive breast cancer and triple-negative breast cancer (TNBC) [5–7], indicating that NAC could offer prognostic information. Moreover, patients who do not achieve pCR may benefit from the opportunity to receive additional adjuvant treatment [8, 9].

With the introduction of multigene assays such as Oncotype DX^®^, the unnecessary use of chemotherapy in estrogen receptor-positive, HER2-negative (ER + HER2-) breast cancer has declined. This subtype relies less on chemotherapy than others, as anti-hormonal therapy provides an effective treatment option. However, in patients with stage II-III ER + HER2- breast caner presenting with high-risk features, such as histologic grade (HG) III, the use of NAC is increasing. Despite this, ER + HER2- breast cancer remains less responsive to chemotherapy compared to other subtypes [10–12], and the likelihood of achieving pCR after NAC remains low in this population [13].

Recent clinical trials have demonstrated that incorporating immune checkpoint inhibitors (ICIs) into NAC enhances the rate of pCR in patients with early-stage, HG III, ER + HER2- breast cancer [14, 15]. In two trials evaluating programmed death-ligand 1 (PD-L1) inhibitors with NAC, the KEYNOTE-756 trial reported a pCR rate of 24.3% with pembrolizumab plus NAC vs. 15.6% with NAC alone (*p* = 0.00005), whereas the CheckMate-7FL trial reported a pCR rate of 24.5% with nivolumab plus NAC vs. 13.8% with NAC alone (*p* = 0.0021). Additionally, an exploratory biomarker analysis from the CheckMate-7FL trial, presented at the *San Antonio Breast Cancer Symposium 2023*, provided evidence supporting the efficacy of nivolumab in combination with NAC based not only on PD-L1 status, a well-established immune marker, but also on stromal tumor-infiltrating lymphocytes (sTIL) levels. In the subgroup with sTIL ≥ 1%– a threshold indicative of sTIL presence and corresponding to the median value among enrolled patients– addition of immunotherapy was associated with a higher pCR rate than NAC alone. Moreover, the benefit of chemoimmunotherapy in achieving pCR increased as the sTIL threshold rose [16]. These findings are consistent with those of a pooled analysis of breast cancer patients treated with NAC, which demonstrated a correlation between sTIL levels and pCR rates, irrespective of clinical subtype [17].

These findings have heightened interest in the clinical benefits of chemoimmunotherapy in this patient population. However, compared with other subtypes, research on identifying subgroups within the ER + HER2- subtype with high pCR rates after NAC or on predictive biomarkers associated with pCR remains limited. To address this gap, we evaluated pCR rates according to sTIL levels and HG in patients with ER + HER2- breast cancer who received NAC, aiming to identify those who may benefit from chemoimmunotherapy and to explore predictive biomarkers.

## Methods

### Patient selection and data collections

**Fig. 1:**
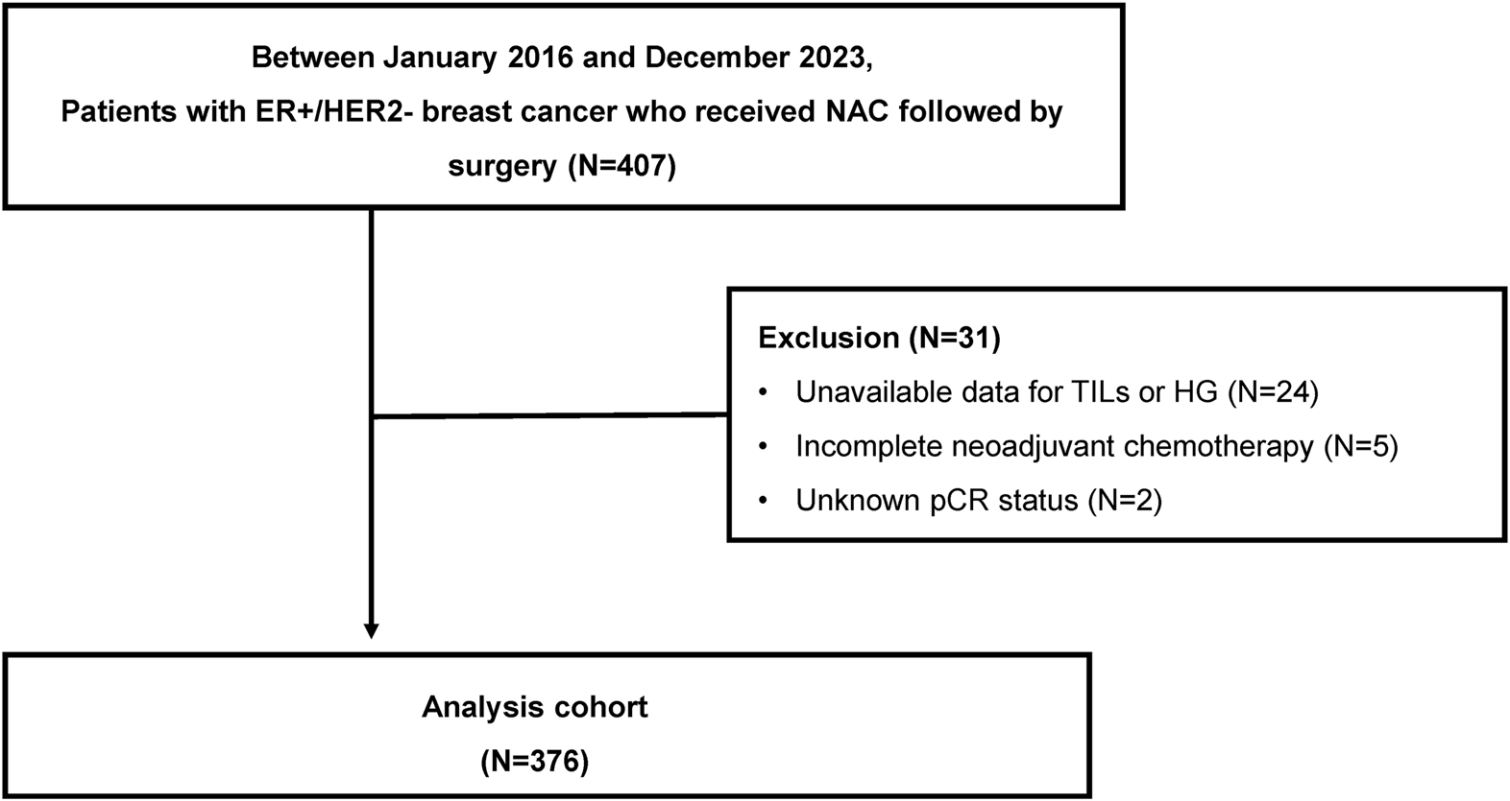
Consort diagram

We retrospectively collected data from patients diagnosed with ER + HER2- breast cancer who received NAC at Gangnam Severance Hospital between January 2016 and December 2023. During this period, 407 ER + HER2- breast cancer patients received NAC at Gangnam Severance Hospital. Of the initial cohort, 31 patients were excluded: 24 due to unavailable data on sTIL levels or HG, five did not complete NAC due to patients’ choice or adverse effects, and two underwent surgery at other institutions, leaving their pCR status unknown. Consequently, 376 patients were eligible for inclusion (Fig. 1).

Clinicopathological data collected from electronic medical records including age at diagnosis, ER and progesterone receptor (PR) expression levels, HG and nuclear grade (NG), Ki67 labeling index (LI), clinical T stage, clinical node positivity, and residual cancer burden (RCB) index, which provided information of residual tumor size and the number of remaining metastatic nodes. All pathological data, with the exception of the RCB index, were obtained biopsied samples prior to NAC administration. Cases were considered ER- and PR-positive if more than 1% of the tumor nuclei in the samples were stained [18]. Additionally, we assessed ER and PR expression through immunohistochemical staining using the modified Allred scoring system [19] and categorized the results into two groups (Allred score (AS) of 1–4 and 5–8) to examine their distribution based on sTIL levels and HG. Positive nuclear Ki67 staining was assessed based on the percentage of positive tumor cells, defined as Ki67 LI. Clinical T stage and nodal status were determined based on baseline magnetic resonance imaging (MRI) findings, with clinical stages following the anatomical stage based on the American Joint Committee on Cancer Guidelines (8th edition).

### Neoadjuvant treatment of this cohort

All patients in this study received anthracycline-based chemotherapy in neoadjuvant phase, with the majority undergoing NAC according to the AC-T regimen. The AC-T regimen consisted of four cycles of doxorubicin (60mg/m^2^ of body-surface area (BSA)) and cyclophosphamide (600mg/m^2^) (AC) administered at three-week intervals, followed by either four cycles of docetaxel (75mg/m^2^) every three weeks (T) or twelve cycles of weekly paclitaxel (80mg/m^2^) (wP). One patient received six cycles of doxorubicin (50mg/m^2^) and docetaxel (75mg/m^2^) (AT) at three-week intervals. In selected patients, carboplatin was administered every three weeks in combination with docetaxel or weekly paclitaxel, based on response evaluation by MRI following completion of the AC regimen or at the discretion of the treating physician. Carboplatin was administered at an area under the plasma concentration-time curve of 5 (AUC 5), which dosing calculated using the Calvert formula [20]. Uncommonly, two patients received trastuzumab (initial dose, 4 mg/kg; maintenance dose, 2 mg/kg) every three weeks in combination with docetaxel after completing the AC regimen. This treatment was guided by the results of a PAM50 study, which classified their tumors as HER2-enriched subtype. Summarized information on NAC is provided in Supplementary Table 1.

No patients in this study received ICIs or endocrine therapy during the neoadjuvant phase. To minimize potential confounding, data from patients who discontinued chemotherapy or required dose reductions below the planned regimen were excluded from the analysis.

### Assessment of stromal tumor-infiltrating lymphocytes (sTIL) and histologic grade (HG)

The levels of sTIL were assessed according to the International TIL Working Group Guidelines [21, 22]. Briefly, the proportion of the stromal area occupied by mononuclear inflammatory cells, such as lymphocytes and plasma cells, was measured, while polymononuclear leukocytes and macrophages were excluded from scoring sTIL within areas of extensive fibrosis, crush artifacts, necrosis, and regressive hyalinization at the tumor border were excluded from the evaluation. The final sTIL score was reported as an average percentage. For the analysis, high sTIL cutoffs were set at 10%, 20%, and 30%, and patients were categorized into high or low sTIL groups based on these thresholds. The high sTIl subgroup included patients with sTIL levels at or above the specified cutoff, whereas the low sTIL subgroup comprised those with sTIL levels below the threshold. For example, with a cutoff value of 20%, patients with sTIL ≥ 20% were assigned to the high sTIL subgroup, while those with sTIL < 20% were classified into the low sTIL subgroup.

HG was classified as grade I, II, and III using the Nottingham grading system [23]; for statistical purpose, grade I/II and grade III were dichotomized.

### Definition of pathological complete response (pCR)

In this study, pCR was defined as the simultaneous absence of residual invasive tumor cells in the breast (breast pCR, ypT0/Tis) and the absence of residual tumor cells in the axilla (axilla pCR, ypN0), collectively designated as ypT0/TisN0. Cases with residual in situ carcinoma without an invasive component in the breast (ypTis) were classified as breast pCR, whereas those with isolated tumor cells in the resected axillary nodes (ypT0(itc+)) were not classified as axillary pCR in accordance with College of American Pathologist (CAP) protocols [24, 25].

### Statistical analysis

The primary objective of this study was to examine the association of pCR rates with sTIL levels and HG. The pCR rates were compared between groups stratified by sTIL and HG status using the chi-square test or Fisher’s exact test, as appropriate. Categorical variables were analyzed similarly. The distribution of continuous variables, such as sTIL levels, were compared using the student’s t-test. When sTIL was reported as a range (e.g., 10–20%), the median value was assigned as the continuous variable for analysis. Multivariable logistic regression analyses were conducted to identify independent predictors of pCR, adjusting for demographic and clinicopathological factors such as age at diagnosis, ER and PR expression status, and clinical T and N stages. Results were reported as odds ratio (OR) with 95% confidence intervals (CIs), with statistical significance set at p-value (*p*) ≤ 0.05. All analyses were performed using SPSS software version 28.0 (SPSS Inc., Chicago, IL, USA) and GraphPad Prism software version 10 (GraphPad Software Inc., MA, USA).

## Results

### Characteristics of the study population

Table 1 shows the demographic and clinicopathological characteristics of 376 patients according to HG. Twenty-seven patients (7.2%) had HG III tumors. Compared with the HG I/II group, the HG III group significantly had larger proportion of low ER cases (ER < 10%; AS ≤ 4). When evaluated using the modified Allred scoring system, 77.8% (21/27) of the tumors in the HG III group had an AS of 5–8, in contrast of 94.6% (330/349) in the HG I/II group (*p* = 0.005). PR expression was also lower in the HG III group (tumors with an AS of 5–8; 29.6% (8/27) in the HG III group vs. 56.4% (197/349) in the HG I/II group; *p* = 0.037).

**Table 1:** Baseline characteristics of patients based on HG

Variables, *N* (%)	HG I/II (*N* = 349)	HG III (*N* = 27)	*p*-value
Age distribution			0.757
≤ 50	230 (65.9)	17 (63.0)	
> 50	119 (34.1)	10 (37.0)	
ER expression			0.005
Allred score 5–8	330 (94.6)	21 (77.8)	
Allred score 1–4	19 (5.4)	6 (22.2)	
PR expression			0.007
Allred score 5–8	197 (56.4)	8 (29.6)	
Allred score 1–4	152 (43.6)	19 (70.4)	
Ki67 LI ^*^			0.037
≥ 14%	39 (54.9)	7 (100)	
< 14%	32 (45.1)	0	
Clinical T stage			0.124
T1	39 (11.2)	1 (3.7)	
T2	177 (50.7)	19 (70.4)	
T3	133 (38.1)	7 (25.9)	
Clinical node positivity			0.708
Positive	26 (7.4)	1 (3.7)	
Negative	323 (92.6)	26 (96.3)	
sTIL, mean (range)	12.9 (5–90)	38.7 (5–95)	< 0.001^#^
sTIL status (cutoff 10%)			< 0.001
High	147 (42.1)	21 (77.8)	
Low	202 (57.9)	6 (22.2)	
sTIL status (cutoff 20%)			< 0.001
High	76 (21.8)	19 (70.4)	
Low	273 (78.2)	8 (29.6)	
sTIL status (cutoff 30%)			< 0.001
High	45 (12.9)	15 (55.6)	
Low	304 (87.1)	12 (44.4)	

^*^Patients with unavailable data were excluded

^#^Student-t test was applied

*Abbreviations* HG, histologic grade; ER, estrogen receptor; PR, progesterone receptor; LI, labelling index; sTIL, stromal tumor-infiltrating lymphocytes

The mean sTIL value in the HG III group was 38.7% (range, 5–95), which was significantly higher than 12.9% (range, 5–90) observed in the HG I/II group (*p* < 0.001). In addition, the proportion of tumors with high sTIL was significantly higher in the HG III group than in the HG I/II group, regardless of the sTIL threshold. Specifically, the proportion of tumors with sTIL ≥ 10% was 77.8% in the HG III group and 42.1% in the HG I/II group (*p* < 0.001). Similarly, the proportion of tumors with sTIL ≥ 20% was 70.4% in the HG III group and 21.8% in the HG I/II group (*p* < 0.001), while the proportion of tumors with sTIL ≥ 30% was 55.6% in the HG III group and 12.9% in the HG I/II group (*p* = 0.001). No notable differences were observed between the two groups concerning the distribution of clinical T stage or clinical node positivity.

### Differences in pCR rates based on HG and sTIL status

**Fig. 2:**
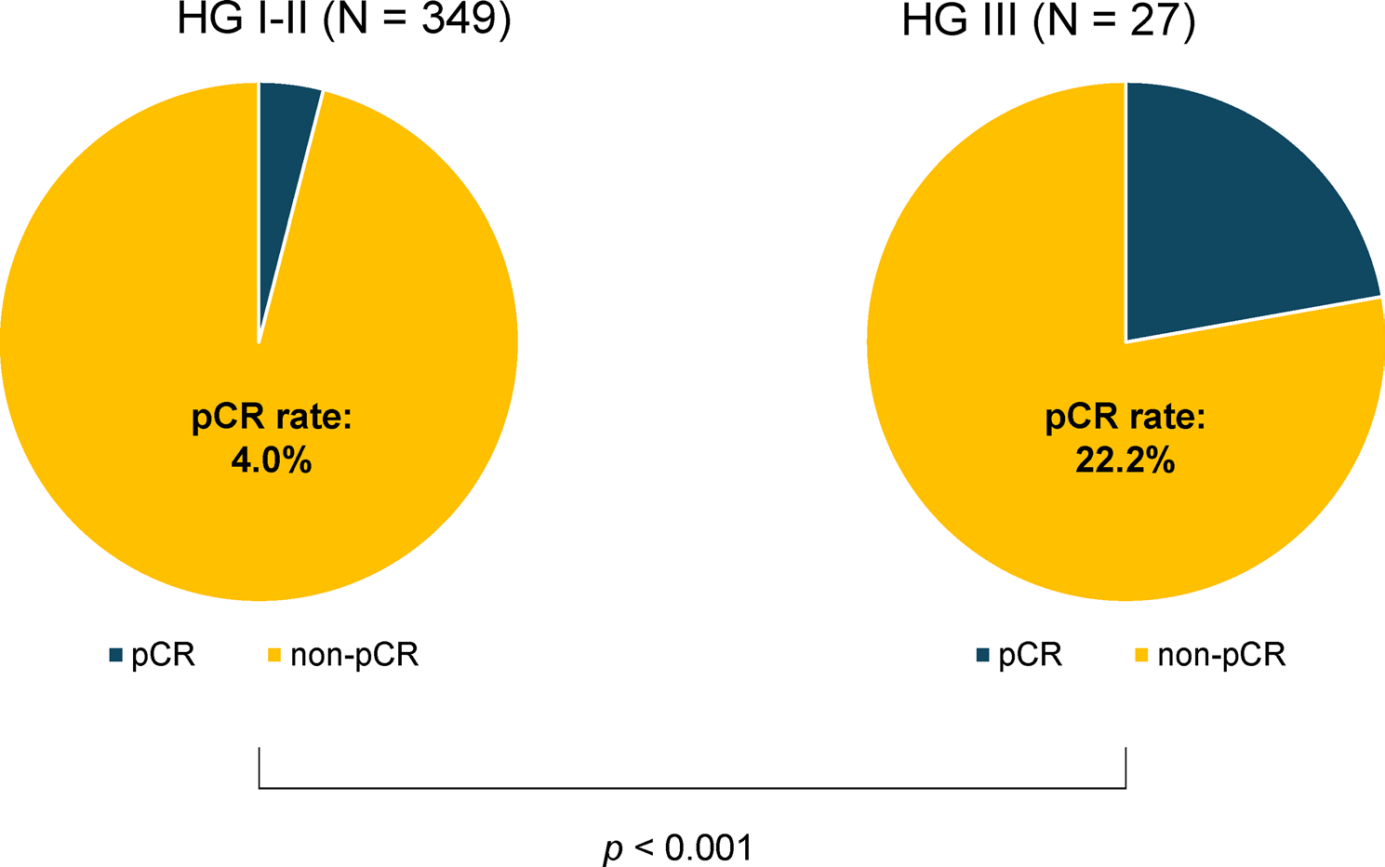
Differences in pathological complete response (pCR) rates in the total cohort by histologic grade (HG). The pCR rate in the HG III group was significantly higher than that in the HG I/II group (22.2% vs. 4.0%; *p* < 0.001)

**Fig. 3:**
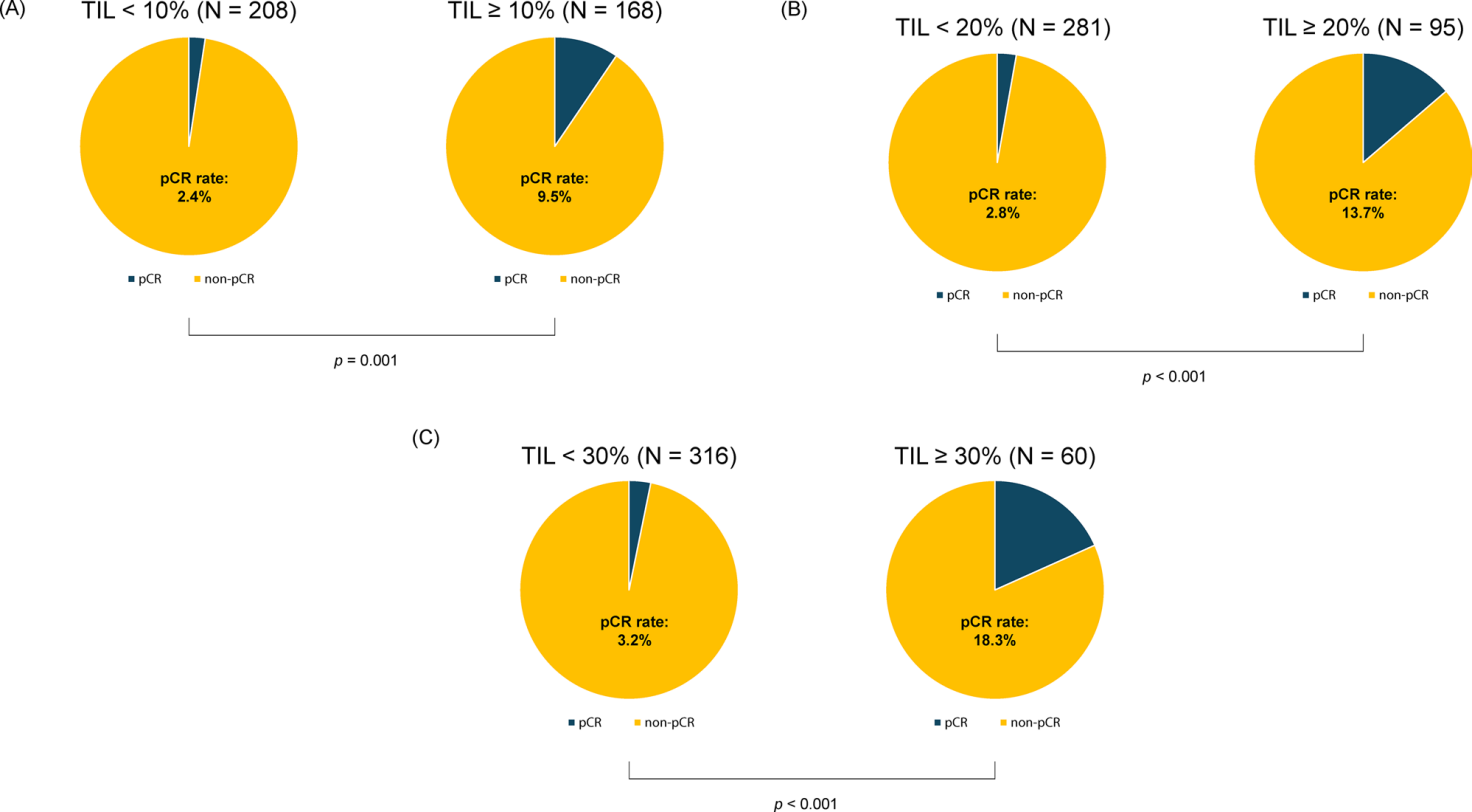
Differences in pathological complete response (pCR) rates in the total cohort based on stromal tumor-infiltrating lymphocytes (sTIL) status. (**A**) With a high sTIL cutoff of 10%, the pCR rate was significantly higher in the high sTIL group compared to the low sTIL group (9.5% vs. 2.4%; *p* = 0.001). (**B**) With a high sTIL cutoff of 20%, the high sTIL group continued to have a significantly higher pCR rate compared to the low sTIL group (13.7% vs. 2.8%; *p* < 0.001). (**C**) With a high sTIL cutoff of 30%, the pCR rate remained significantly higher in the high sTIL group compared to the low sTIL group (18.3% vs. 3.2%; *p* < 0.001)

The HG III group achieved a significantly higher rate of pCR compared to the HG I/II group (22.2% vs. 4.0%; *p* < 0.001, Fig. 2). Next, we assessed the pCR rates across the entire patient cohort based on sTIL levels. When the high sTIL cutoff was set at 10%, the pCR rate was significantly higher in the high sTIL group (sTIL ≥ 10%) compared to 1.9% in the low sTIL group (sTIL < 10%) (9.5% vs. 2.4%; *p* = 0.001, Fig. 3A). This trend persisted even at higher cutoff values. Specifically, at a 20% cutoff, the pCR rate was 13.7% in the high sTIL group (sTIL ≥ 20%) versus 2.8% in the low sTIL group (sTIL < 20%) (*p* < 0.001, Fig. 3B), and at a 30% cutoff, the pCR rate was 18.3% in the high sTIL group (sTIL ≥ 30%) compared to 3.2% in the low sTIL group (sTIL < 30%) (*p* = 0.001, Fig. 3C).

### Subgroup analysis to assess the difference in pCR rates by sTIL status within each HG group

**Fig. 4:**
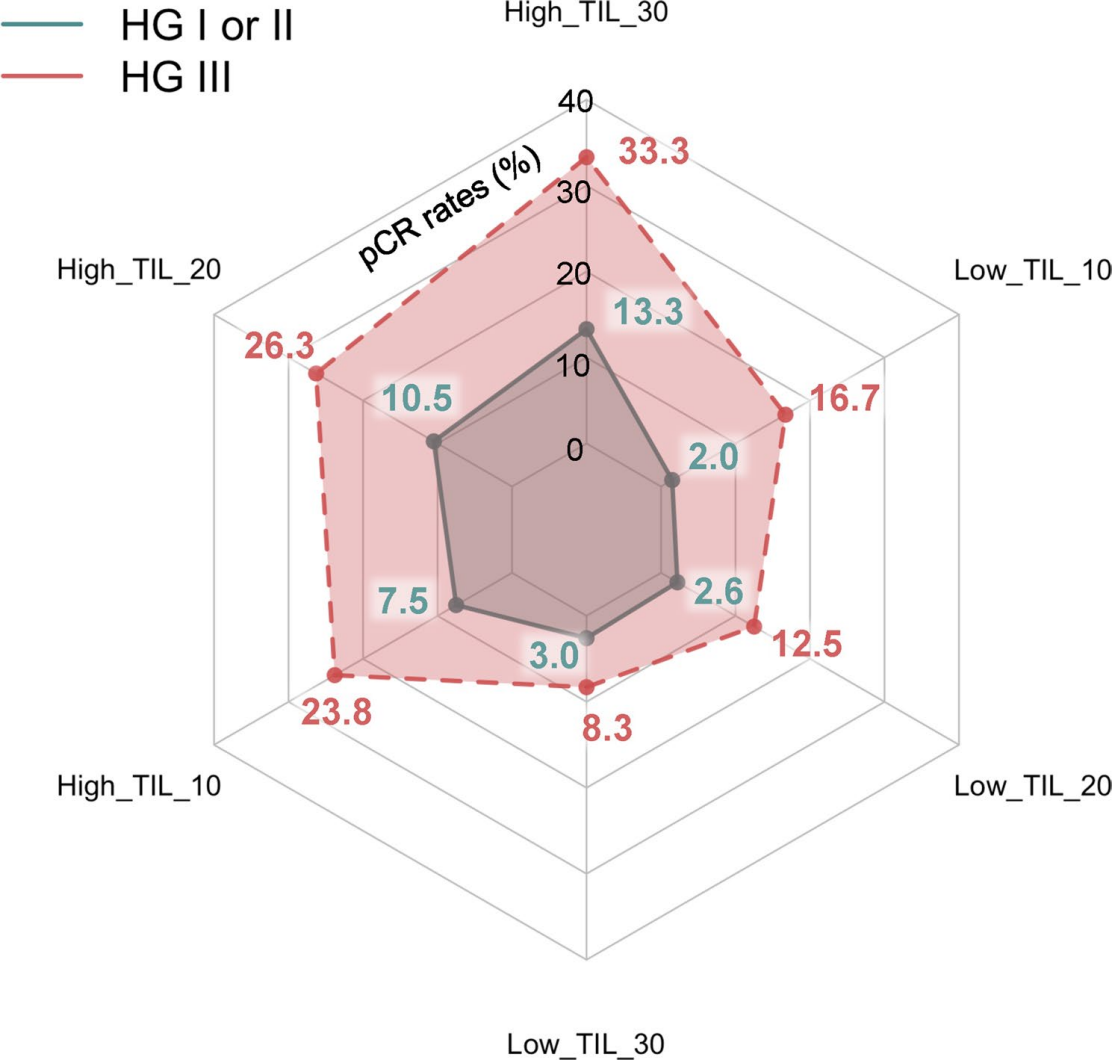
Radar chart illustrating the pathological complete response (pCR) rates based on stromal tumor-infiltrating lymphocytes (sTIL) and histologic grade (HG). The blue line represents the HG I/II group, while the red line corresponds to the HG III group, with each vertex on the hexagon indicating pCR rates according to different sTIL cutoffs. In the HG I/II group, pCR rates were statistically significantly higher in the high sTIL group compared to the low sTIL group across all cutoffs. Specifically, at a sTIL cutoff of 10%, the pCR rate was 7.5% in the high sTIL subgroup versus 2.0% in the low sTIL subgroup (*p* = 0.005). At a 20% cutoff, the pCR rate was 10.5% in the high sTIL subgroup compared to 2.6% in the low sTIL subgroup (*p* = 0.001). At the 30% cutoff, the high sTIL subgroup had a pCR rate of 13.3%, while the low sTIL subgroup had a rate of 3.0% (*p* = 0.001). In contrast, within the HG III group, while the high sTIL subgroup showed a trend toward higher pCR rates than the low sTIL subgroup, these differences were not statistically significant. Specifically, at a sTIL cutoff of 10%, the pCR rate was 23.8% in the high sTIL subgroup and 16.7% in the low sTIL subgroup (*p* > 0.999). At a 20% cutoff, the pCR rate in the high sTIL subgroup was 26.3% compared to 12.5% in the low sTIL subgroup (*p* = 0.633). For the 30% cutoff, the high sTIL subgroup had a pCR rate of 33.3%, while the low sTIL subgroup had a rate of 8.3% (*p* = 0.182)

To assess the relationship between pCR rates, sTIL levels, and HG, a subgroup analysis was conducted with each HG group to compare pCR rates according to sTIL status (Fig. 4). The highest pCR rate was observed in patients with HG III and sTIL ≥ 30%, at 33.3% (5 of 15). When stratified by an sTIL cutoff of 30% within the HG III group, the difference in pCR rates between the high and low sTIL subgroups was 25.0% (33.3% vs. 8.3%). Although not statistically significant, the pCR rate in the HG III group was consistently higher in the high sTIL subgroup than in the low sTIL subgroup, irrespective of the sTIL cutoff applied. Specifically, the pCR rate was 23.8% (5/21) in patients with sTIL ≥ 10% and 16.7% (1/6) in those with sTIL < 10% (*p* > 0.999). When the sTIL cutoff was set at 20%, the pCR rate was 26.3% (5/19) in the high sTIL subgroup and 12.5% (1 of 8) in the low sTIL subgroup (*p* = 0.633). As previously mentioned, at the 30% cutoff, the pCR rate was 33.3% (5/15) in the high sTIL subgroup and 3.0% (9/304) in the low sTIL subgroup (*p* = 0.182).

### Validation of the levels of sTIL and HG as independent predictors of pCR

To evaluate the clinical importance of sTIL levels and HG as independent predictors of pCR, we performed multivariable logistic regression analyses. No significant associations were observed between pCR and other factors besides sTIL levels and HG in the univariable analysis; thus, a multivariate-adjusted model was applied to determine whether the levels of sTIL and HG independently predict pCR. This model adjusted for demographic and clinicopathological variables, including age at diagnosis, ER and PR expression status, clinical T stage, and clinical node positivity.

Table 2 demonstrates the results of the multivariate-adjusted model, which analyzed the associations between sTIL status and pCR as well as HG and pCR. With a high sTIL cutoff set at 10%, both high sTIL status and HG III were identified as independent predictors. However, at cutoffs of 20% and 30%, with high sTIL status remained significantly associated with pCR, the association between HG and pCR was observed but did not reach statistical significance.

**Table 2:** Multivariate-adjusted analyses for the association between pCR and sTIL levels, HG

Variables	Multivariate		
	Adjusted OR^*^	95% CIs	*p*-value
HG III	3.9	1.2–12.6	0.025
HG I/II	Ref.		
sTIL ≥ 10%	3.7	1.1–12.8	0.037
sTIL < 10%	Ref.		
HG III	3.2	0.97–10.7	0.057
HG I/II	Ref.		
sTIL ≥ 20%	3.71	1.2–11.3	0.021
sTIL < 20%	Ref.		
HG III	3.2	0.95–10.7	0.06
HG I/II	Ref.		
sTIL ≥ 30%	4.1	1.3–12.8	0.014
sTIL < 30%	Ref.		

* Adjusted for age at diagnosis, ER expression, PR expression, clinical T stage, and clinical node involvement

*Abbreviations* OR, odds ratios; CIs, confidence intervals; HG, histologic grade; sTIL, stromal tumor-infiltrating lymphocytes

## Discussion

In this study, we assessed pCR rates in relation to sTIL levels and HG in patients with ER + HER2- breast cancer treated with NAC. Patients with HG III tumors had higher pCR rates than did those with HG I/II tumors. Similarly, patients with high sTIL levels had higher pCR rates than did those with low sTIL levels. In the HG I/II group, the pCR rate was consistently higher in patients with high sTIL levels, irrespective of a cutoff value. Although no statistically significant difference was observed in the HG III group, a similar trend was observed. Multivariate-adjusted analysis confirmed that increasing sTIL levels and HG III were independent predictors of pCR. While HG III exhibited marginal significance when the high sTIL cutoff was set at 10%, the trend of higher pCR rates in HG III tumor persisted, and a high sTIL status remained an independent predictor of pCR across all cutoffs.

In ER + HER2- breast cancer, where tumorigenesis is driven by the estrogen-dependent pathway, anti-estrogen therapy is the cornerstone of treatment, and the prognosis is generally more favorable than that of other subtypes [26–28]. However, in subgroups with high-risk features such as node-positive disease, an increased risk of recurrence necessitates treatment intensification [29]. In particular, the majority of patients with ER + HER2- breast cancer remain candidates for chemotherapy, resulting in a reduced pCR rate [11, 12]. Given that extensive residual cancer is associated with poorer outcomes, even in this subtype [13], enhancing the response to chemotherapy is crucial, an identifying the influence of chemotherapy is also important.

Immunotherapy has emerged as a significant research focus among novel approaches in cancer treatment. Despite breast cancer traditionally being considered less immunogenic than other solid tumors, thereby constraining early research, recent studies have investigated immunotherapy in breast cancer, notably the use of PD-L1 inhibitors in TNBC [30]. This observation has prompted research into whether adding immunotherapy to NAC could improve pCR rates in these subgroups. The KEYNOTE-756 and CheckMate-7FL trials demonstrated that adding PD-L1 inhibitors (pembrolizumab and nivolumab, respectively) to NAC significantly improved pCR rates in patients with early-stage, high-risk, ER + HER2- breast cancer [14, 15]. Most participants in these studies had HG III tumors. Although CheckMate-7FL allowed the inclusion of patients with HG II tumors and low ER expression, their enrollment was minimal. ER + HER2- breast cancer with HG III features shows more aggressive clinical behavior than HG I/II tumors, characterized by larger tumor size, higher nodal involvement, and increased proliferative index, which correlate with worse prognosis and the need for more intensive therapy [31–33]. However, transcriptional profiling reveals downregulation of ER signaling and upregulation of immune-related pathways, suggesting that this subgroup might have a more immunogenic profile within the ER + HER2- breast cancer group [34, 35]. Immunogenicity may enhance the response to chemotherapy and ICIs. In addition, a meta-analysis showed that among ER + HER2- breast cancer who received NAC, the pCR rate was higher in the HG III group than in the HG I/II group [5]. When these findings were integrated with the results of our study, HG emerged as a significant predictor of pCR following NAC in ER + HER2- breast cancer.

Stromal tumor-infiltrating lymphocytes (sTIL) are important indicators of the immune response and prognosis in breast cancer; however, their predictive and prognostic roles in ER + HER2- breast cancer remain unclear [17, 36, 37]. The International TILs Working Group has proposed an sTIL cutoff of 50% of define lymphocyte-predominant breast cancer, although their guidelines acknowledge that the prognostic and predictive value of sTIL is most evident in TNBC and HER2-positive breast cancer [21]. Indeed, prior studies using this cutoff have demonstrated limited clinical significance of sTIL status for predicting neoadjuvant response or oncologic outcomes in patients with ER + HER2- breast cancer compared with other subtypes [17, 38, 39]. Although some ER + HER2- tumors exhibit elevated immune-related gene expression profiles similar to TNBC [40–43], research identifying clinically meaningful sTIL thresholds that can be readily assessed through immunohistochemical staining remains insufficient. This scarcity of data may partly reflect the lower median sTIL value typically observed in ER + HER2- breast cancer relative to other subtypes [17, 44]. Landmark trials assessing the efficacy of ICIs in ER + HER2- breast cancer, including the I-SPY2 and KEYNOTE-756 trials, did not incorporate exploratory analyses of sTIL [14, 45]. Moreover, while the CheckMate-7FL trial evaluated the predictive value of sTIL, it applied relatively low threshold values [15]. In our analysis, although more than half of the enrolled patients had sTIL levels below 10%, the median sTIL level for the entire cohort was 14.8%, increasing to 38.7% among patients with HG III tumors– values notably higher than those reported in previous studies. Given these findings, we explore multiple sTIL thresholds– 10%, 20%, and 30%– to determine clinically relevant cutoff points predictive of treatment response. The pCR rate was significantly higher in the high sTIL subgroup than in the low sTIL subgroup, with the difference becoming more pronounced at higher sTIL thresholds. These findings align with previous evidence suggesting that the tumor immune microenvironment plays a role in chemotherapy response, even in ER + HER2- breast cancer [46, 47]. Moreover, this supports the notion that combining immunotherapy with NAC in ER + HER2- breast cancer with high sTIL levels could potentially enhance treatment response. Consequently, although further studies are needed to clarify the relationship between sTIL and other immune markers such as PD-L1, our findings support the potential benefit of combining immunotherapy with chemotherapy to improve treatment outcomes in patients with ER + HER2- breast cancer characterized by high sTIL levels.

Our study offers valuable insights into the relationship between HG, sTIL levels, and response to NAC in ER + HER2- breast cancer, an area that remains relatively underexplored. Conducted as a single-center study, centralized surgical procedures and pathological assessments were employed to minimize potential biases inherent in the data. Furthermore, by focusing on the relatively underrepresented Asian population, this study provides critical evidence that could guide future research and serve as a reference for subsequent analyses. However, this study has several limitations. First, its retrospective study design introduces potential selection bias and limits the ability to fully account for confounding factors, reflecting the intrinsic constraints of this study. Additionally, the relatively small sample size poses a significant limitation. For instance, within the HG III group, although a trend towards higher pCR rates in the high sTIL subgroup was observed, it did not reach statistical significance. This lack of significance is likely attributable to the limited number of patients in this subgroup, reducing statistical power and potentially leading to analytical error. Moreover, an important limitation of this study is the lack of evaluation of survival outcomes, such as event-free survival stratified by pCR status. Prior studies have suggested that high sTIL levels may be associated with worse outcomes in ER + HER2- breast cancer [38, 43]. As a result, the clinical significance of sTIL levels of HG in this subtype remains incompletely defined. Future studies should incorporate in-depth genomic analyses to explore the tumor and immune components associated with both favorable and unfavorable outcomes following pCR in ER + HER2- breast cancer.

## Conclusions

Our study demonstrated that among ER + HER2- breast cancer patients treated with NAC, those with tumors exhibiting high sTIL levels achieved higher pCR rates, an effect that appeared more pronounced in HG III tumor. Although further refined research and validation are necessary, our findings lay the groundwork for tailored treatment in early-stage, high-risk, ER + HER2- breast cancer patients who might derive clinical benefits from combined immunotherapy and chemotherapy.

## Electronic supplementary material

Below is the link to the electronic supplementary material.

Supplementary Material 1

## Data Availability

The data that support the findings of this study contain clinical outcomes for which institutional review board (IRB) approved was required before the analysis. Therefore, these data are not publicly available. The data will be provided to authorized researchers who have obtained IRB approval from Gangnam Severance Hospital, Yonsei University, Seoul, Republic of Korea. For data access requests, please contact the corresponding author: S.G. Ahn (asg2004@yuhs.ac).

## Abbreviations

ER: Estrogen receptor
HER2: Human epidermal growth factor receptor 2
HG: Histologic grade
sTIL: Stromal tumor-infiltrating lymphocytes
NAC: Neoadjuvant chemotherapy
ICIs: Immune checkpoint inhibitors
pCR: Pathological complete response
