# Supplementary material for: Pathological complete response, histologic grade, and level of stromal tumor-infiltrating lymphocytes in ER + HER2- breast cancer
Source: Breast Cancer Res. 2025 Mar 20;27:42. doi: 10.1186/s13058-025-01999-7 (PMC11927358; doi:10.1186/s13058-025-01999-7)
Supplement: Supplementary file 1 — Supplementary Material 1 [file 13058_2025_1999_MOESM1_ESM.docx]

Supplementary Table 1. Distribution of neoadjuvant chemotherapy in this cohort

| Regimen | N (%) |
| --- | --- |
| AC-T | 181 (48.1) |
| AC-wP | 188 (50.0) |
| AT | 1 (0.3) |
| AC-T plus C | 4 (1.1) |
| AC-TH | 2 (0.5) |
| Total | 376 |

Abbreviations, AC-T, adriamycin and cyclophosphamide followed by docetaxel; AC-wP, adriamycin and cyclophosphamide followed by weekly paclitaxel; AT, adriamycin and docetaxel; C, carboplatin; H, trastuzumab
